# Structural features distinguishing infectious *ex vivo* mammalian prions from non-infectious fibrillar assemblies generated *in vitro*

**DOI:** 10.1038/s41598-018-36700-w

**Published:** 2019-01-23

**Authors:** Cassandra Terry, Robert L. Harniman, Jessica Sells, Adam Wenborn, Susan Joiner, Helen R. Saibil, Mervyn J. Miles, John Collinge, Jonathan D. F. Wadsworth

**Affiliations:** 1 MRC Prion Unit at UCL, UCL Institute of Prion Diseases, 33 Cleveland Street, London, W1W 7FF UK; 2 School of Chemistry, University of Bristol, Bristol, BS8 1TS UK; 3 Institute of Structural and Molecular Biology, Department of Biological Sciences, Birkbeck College, University of London, Malet Street, London, WC1E 7HX UK; 4 School of Physics, H.H. Wills Physics Laboratory, University of Bristol, Tyndall Avenue, Bristol, BS8 1TL UK; 5 Present Address: London Metropolitan University, North Campus, Holloway Road, London, N7 8DB UK; 6 Present Address: King’s Centre for Stem Cells & Regenerative Medicine, King’s College London, Guy’s Campus, London, SE1 9RT UK

## Abstract

Seeded polymerisation of proteins forming amyloid fibres and their spread in tissues has been implicated in the pathogenesis of multiple neurodegenerative diseases: so called “prion-like” mechanisms. While *ex vivo* mammalian prions, composed of multichain assemblies of misfolded host-encoded prion protein (PrP), act as lethal infectious agents, PrP amyloid fibrils produced *in vitro* generally do not. The high-resolution structure of authentic infectious prions and the structural basis of prion strain diversity remain unknown. Here we use cryo-electron microscopy and atomic force microscopy to examine the structure of highly infectious PrP rods isolated from mouse brain in comparison to non-infectious recombinant PrP fibrils generated *in vitro*. Non-infectious recombinant PrP fibrils are 10 nm wide single fibres, with a double helical repeating substructure displaying small variations in adhesive force interactions across their width. In contrast, infectious PrP rods are 20 nm wide and contain two fibres, each with a double helical repeating substructure, separated by a central gap of 8–10 nm in width. This gap contains an irregularly structured material whose adhesive force properties are strikingly different to that of the fibres, suggestive of a distinct composition. The structure of the infectious PrP rods, which cause lethal neurodegeneration, readily differentiates them from all other protein assemblies so far characterised in other neurodegenerative diseases.

## Introduction

Mammalian prions are infectious agents composed principally or entirely of multichain assemblies of misfolded, host-encoded prion protein (a glycosylphosphatidylinositol (GPI)-anchored cell surface glycoprotein), some of which acquire protease-resistance and are classically designated as PrP^Sc^ ^1–3^. Prions propagate by means of seeded protein polymerization, a process that involves the recruitment of PrP monomers to fibrillar assemblies followed by fission of the polymer to produce more “seeds”. Different prion strains can propagate in the same inbred host to produce different disease phenotypes and appear to be encoded by distinct PrP conformations and assembly states^1–3^. It is now clear that elucidating the molecular processes involved in mammalian prion propagation and strain diversity will be of major relevance to understanding other human neurodegenerative diseases (including Alzheimer’s and Parkinson’s disease) where seeded polymerisation or “prion-like” propagation of other protein assemblies is now a major research focus^3–7^. To date, despite enormous international effort, the high resolution structure of an infectious mammalian prion remains unknown. Consequently, protein assemblies in other diseases cannot yet be classified as “prion-like” according to defined structural criteria. In this context, it is important that animal models of other neurodegenerative diseases involving propagation and spread of assemblies of different proteins including tau, amyloid-β and α-synuclein do not generally result in lethal neurodegeneration, implying that the basic architecture of mammalian prions may be unique and critical to their ability to act as lethal pathogens^3^.

Progress in understanding prion structure has been severely hindered by the difficulty of isolating relatively homogeneous prion particles from infected tissue and unequivocally correlating infectivity with composition and structure. However, we recently developed new methods for isolating exceptionally pure, high-titre infectious prion preparations from mouse brain and showed that pathogenic PrP in these preparations is assembled into rod-like assemblies (PrP rods) that faithfully transmit prion strain-specific phenotypes when inoculated into mice^8^. Subsequently, using sensitive cell culture infectivity assays we established that the PrP rods are intrinsically infectious and that changing the number of aggregates into which the rods are distributed proportionally changes the number of infectious units available to cells at inoculation^9^. Electron tomography of negatively-stained prion rods from multiple prion strains revealed a common three-dimensional architecture comprising a pair of short, intertwined fibres, each with a double helical repeating substructure, separated by a distinct gap of 8–10 nm in width^9^. Hierarchical assembly of PrP into a paired fibre structure (20 nm in width) appears to be a key feature of infectious prions and contrasts markedly with non-infectious amyloid PrP fibrils generated *in vitro* from bacterially-expressed recombinant PrP which consist of only a single fibre (10 nm wide) composed of a double helical arrangement of two protofilaments^9,10^.

Previously we hypothesised that some of the PrP N-linked glycans (present in the *ex vivo* PrP rods but not in recombinant PrP fibrils) may be involved in linking the paired fibres of the PrP rods and might reside predominantly in the 8–10 nm central gap of the rod^9^. However, the use of negative stain in our previous studies precluded the ability to visualise material in this central region of the rod. Accordingly, in this study we have now examined infectious *ex vivo* PrP rods and non-infectious recombinant PrP fibrils by cryo-electron microscopy (cryo-EM) and atomic force microscopy (AFM) using unstained samples. Images collected show that negative stain makes little difference to the overall dimensions of these assemblies and confirms their key structural differences. AFM imaging and concurrent quantitative measurement of the adhesion interaction with a silicon tip shows unequivocal evidence for biological material in the central 8–10 nm gap of the infectious PrP rods, which now formally excludes the possibility that the paired fibre assembly of the PrP rods is an artefact of staining methods. This central material has an irregular topography and adhesive properties that are significantly different to that of the outer surface of the paired fibres of the PrP rod, consistent with the idea that N-linked glycans may be fulfilling a key structural role^9^. The first cryo-EM images of unstained, high titre, *ex vivo* infectious PrP rods formed from wild-type PrP shown here, allied with significant new AFM findings, now defines the configuration of the authentic infectious prion assembly state that should be targeted in future high resolution structural studies of mammalian prions.

## Results

### Structural features of non-infectious recombinant PrP fibrils

**Figure 1:**
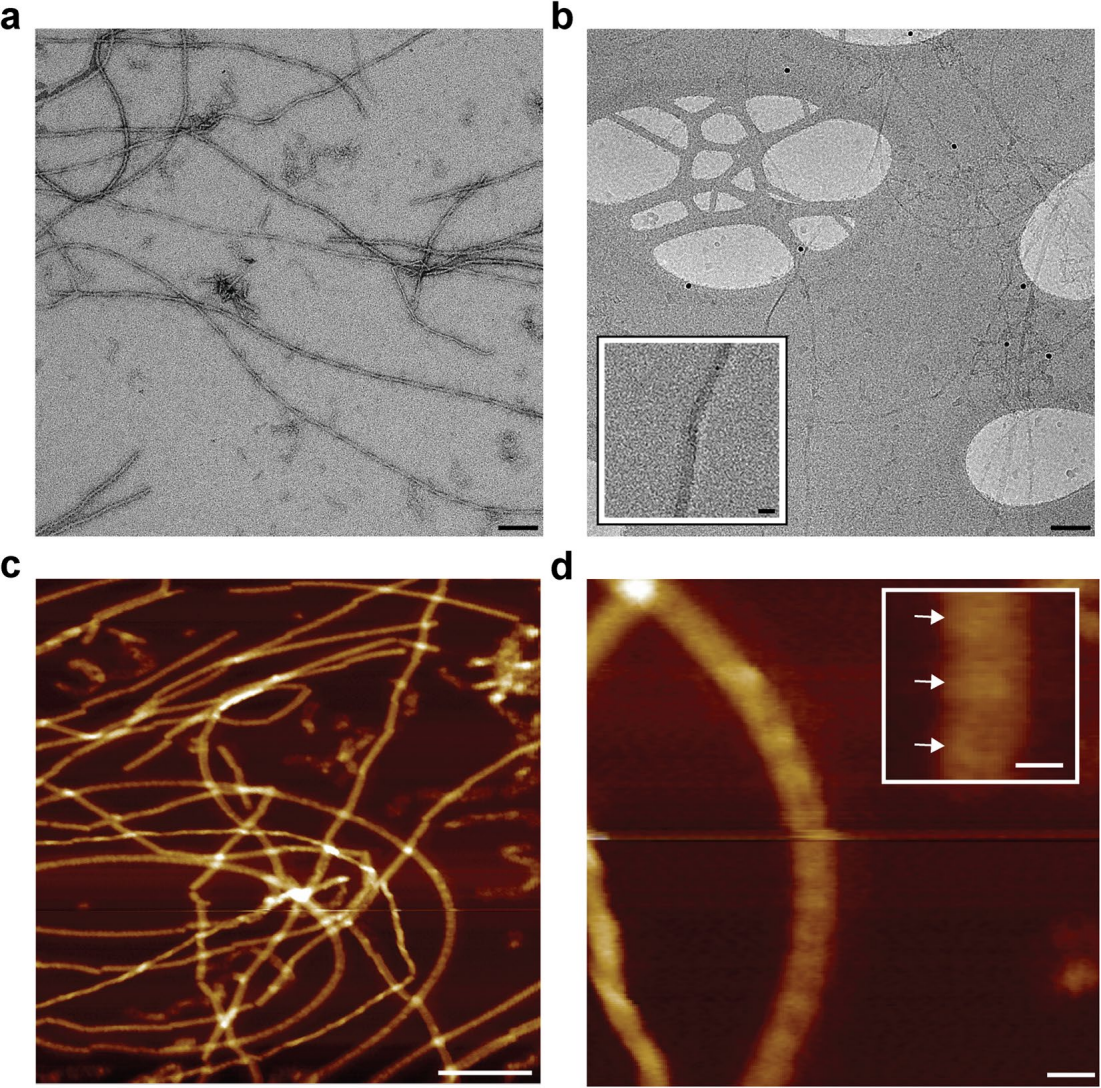
Non-infectious recombinant PrP fibrils imaged by negative stain EM, cryo-EM and AFM. (**a**) EM image of fibrils stained with NanoW at pH 6.8. (**b**) Cryo-EM image of unstained fibrils in vitreous ice. The inset shows a magnified image of one fibril. Protein-A gold 10 nm were added to this sample as fiducial markers. (**c**) 1 µm × 1 µm scan showing height images obtained by AFM. (**d**) An enlarged image of one fibril from panel c with inset showing repeating structure along the fibril length (denoted by arrows) at 7.1 ± 0.8 nm intervals. Scale bars, 100 nm main panels a, b and c, 10 nm, main panel d and inset panel b, 5 nm, inset panel d.

**Figure 2:**
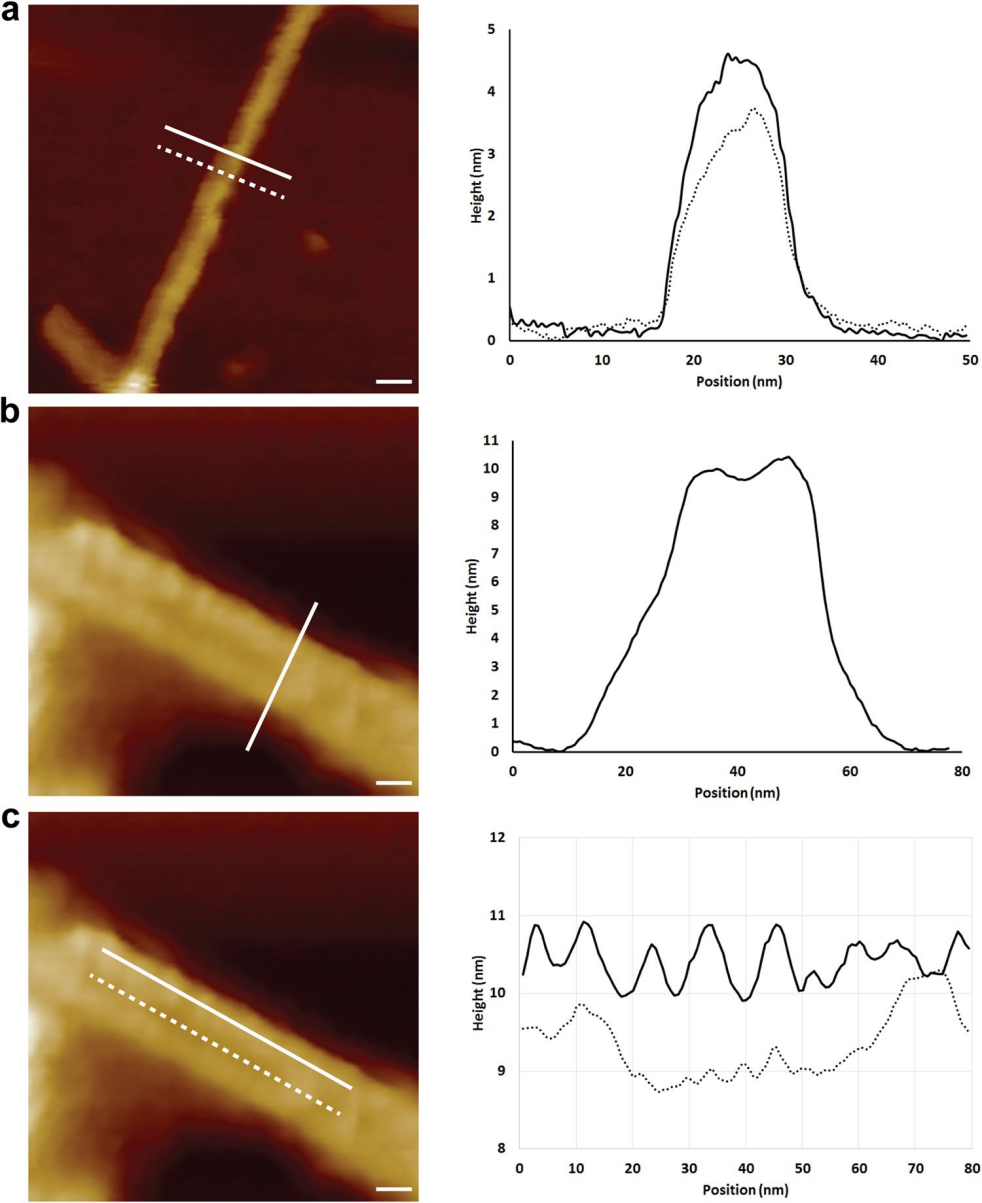
Non-infectious recombinant PrP fibrils and infectious RML PrP rods imaged with high resolution AFM tips. (**a**–**c**) Representative 2D surface topography images. (**a**) Recombinant fibrils have an average width of 10.4 ± 1.2 nm and an average height of 4.0 ± 0.8 nm. The graph plots height profiles at the positions of the solid and dotted lines. (**b**) RML prion rods have a fibre height of 9.2 ± 0.5 nm and an overall width of 26.5 ± 0.3 nm. The height of the central gap material is 7.4 ± 0.8 nm. (**c**) RML prion rods have repeating structure at 6.3 ± 0.4 nm intervals along the length of the fibres (solid line) and have material with a more irregular structure down the 8–10 nm central gap of the rod (dotted line) that separates the paired fibres. The tip broadening artefacts displayed in the raw profiles are removed through deconvolution for the calculation of dimensions given above and in Table 1. Scale bars, 10 nm.

**Figure 3:**
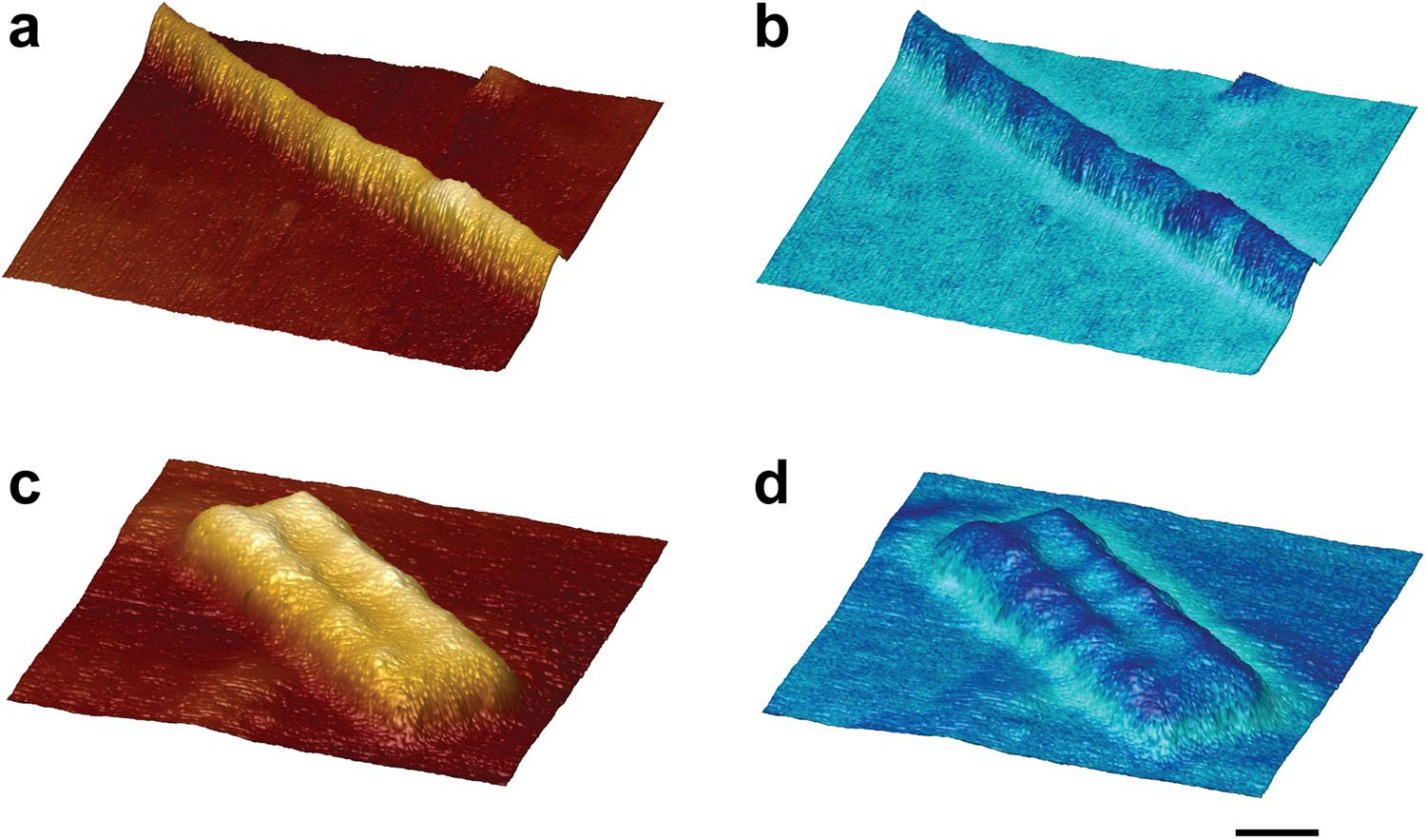
3D-imaging of non-infectious recombinant PrP fibrils and infectious RML PrP rods by AFM. 3D surface renderings of (**a**,**b**) non-infectious recombinant PrP fibrils and (**c**,**d**) infectious RML PrP rods. (**b**,**d**) show the 3D surface renderings with the adhesive interaction force measured by the cantilever tip overlaid as a blue colour-scale (light to dark representing high to low adhesive force). Scale bar, 10 nm.

Non-infectious recombinant mouse PrP fibrils generated from bacterially expressed mouse PrP are composed of two intertwined protofilaments with a subunit repeat of ~6 nm when imaged in 2D and 3D by negative stain EM and electron tomography^9,10^. The fibrils appear as single fibres that are typically several micrometres long and have a width of ~10 nm (Fig. 1a). Here we have examined fibrils formed from full length mouse PrP in their native fully hydrated state by cryo-EM (Fig. 1b) and also by AFM after drying onto mica (Figs 1c,d and 2a). By both methods the morphology and the dimensions of the fibrils were entirely consistent with the negative stain EM images. In particular, a fibril width of ~10 nm was congruent with all three methods (Table 1). AFM provided determination of the height of fibrils as 4.0 ± 0.8 nm (Table 1, Figs 1c and 2a), and width:height ratios were consistent across all of the recombinant PrP fibrils examined. AFM was also used to concurrently measure the surface topography and corresponding adhesion interaction forces between the surface of the fibril and silicon tip. The fibrils were relatively smooth (Figs 1c,d, 2a and 3a) but showed variable adhesive force interactions along their length (Fig. 3b) with a periodicity of 7.1 ± 0.8 nm which corresponds well to the 6 nm repeating subunit density seen by negative stain EM^9,10^. To enable accurate comparison between adhesive data collected on different PrP assemblies the adhesive forces were normalised as a percentage of the adhesive interaction between the silicon tip and exposed mica in each image, as the exposed mica is a common background feature to all samples. All adhesive forces measured were below 1 nN (Table 2). For the recombinant PrP fibril the average adhesive interaction was measured to be 66% of that measured for mica (Table 2) with the periodic variations along their length being ± 19% about the average.

**Table 1:** Dimensions of non-infectious recombinant PrP fibrils and infectious RML prion rods by EM and AFM.

Sample	Method	Width mean ± SD (nm)	Length mean ± SD (nm)	Height mean ± SD (nm)
Recombinant PrP fibrils	Negative stain EM (n = 18 fibrils)	10.4 ± 1.2	>700	—
	Cryo-EM (n = 18 fibrils)	9.3 ± 0.8	>700	—
	AFM (n = 6 fibrils)	11.3 ± 2.2	>700	4.0 ± 0.8
*Ex vivo* RML prion rods	Negative stain EM (n = 18 rods)	20.2 ± 1.9*	127.5 ± 41.3	
	Cryo EM (n = 18 rods)	19.4 ± 2.3^§^	118.3 ± 23.8	
	AFM (n = 8 rods)	26.5 ± 0.3^#^	111.5 ± 17.4	9.2 ± 0.5^$^

^*^Central gap width 9.1 ± 1.4 nm.

^§^Central gap width 8–10 nm.

^#^Central gap width 8.0 ± 0.9 nm.

^$^Central gap height of 7.4 ± 0.8 nm.

**Table 2:** Adhesion force interaction measurements of RML prion rods and recombinant PrP fibrils using AFM.

Sample	Adhesion Force Interaction (nN) Mean ± SD		
	Mica Surface	Fibre surface	Central gap
RML prion rods	0.69 ± 0.07 (*n* = 90)	0.49 ± 0.11 (*n* = 8 *rods*; 242 *total measurements*)^*,†^	0.87 ± 0.08 (*n* = 8 *rods*; 105 *total measurements*)^*,†^
Recombinant PrP fibrils	0.82 ± 0.11 (*n* = 160)	0.54 ± 0.16 (*n* = 6 *fibrils;* 183 *total measurements*)*	N/A

*Means were generated from multiple measurements (>10) made along the length of each individual object (8 RML PrP rods or 6 recombinant PrP fibrils).

^†^Difference in the mean values between the fibre surface and the central gap of the RML rods is statistically significant (p < 0.0001, unpaired t test with Welch correction).

N/A, not applicable.

### Structural features of infectious prion rods

**Figure 4:**
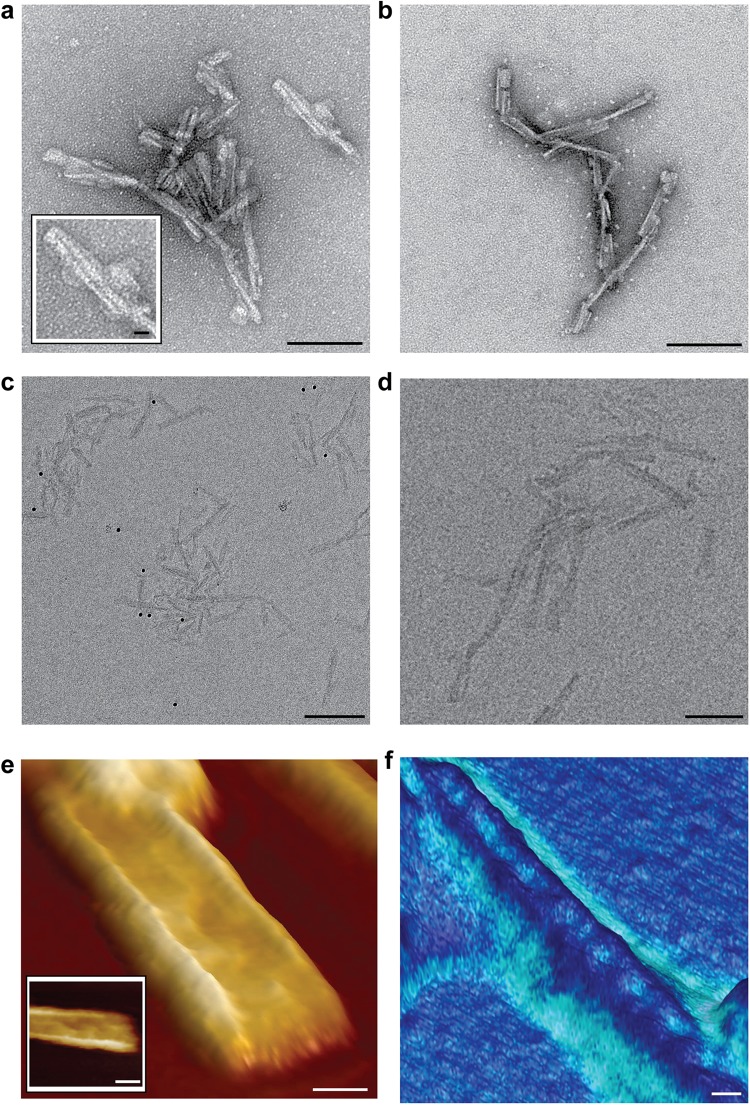
Infectious RML prion rods imaged by negative stain EM, cryo-EM and AFM. (**a**,**b**) Negative stain EM images using (**a**) uranyl acetate at pH 4 and (**b**) NanoW at pH 6.8. (**c**,**d**) Cryo-EM images. (**e**) 140 nm × 140 nm AFM scan revealing the presence of material in the central gap of the rod which appears to link the paired fibres. Main panel 3D image, inset, 2D image. (**f**) AFM surface topography (height) adhesion force mode showing that material in the central gap region of the rod interacts more strongly with the scanning tip (exerts a greater adhesive force) than the rod fibres (light to dark representing high to low adhesive force). Scale bars, 100 nm main panels a, b and d, 200 nm panel c, 10 nm main panels e and f and insets in panels a and e.

**Figure 5:**
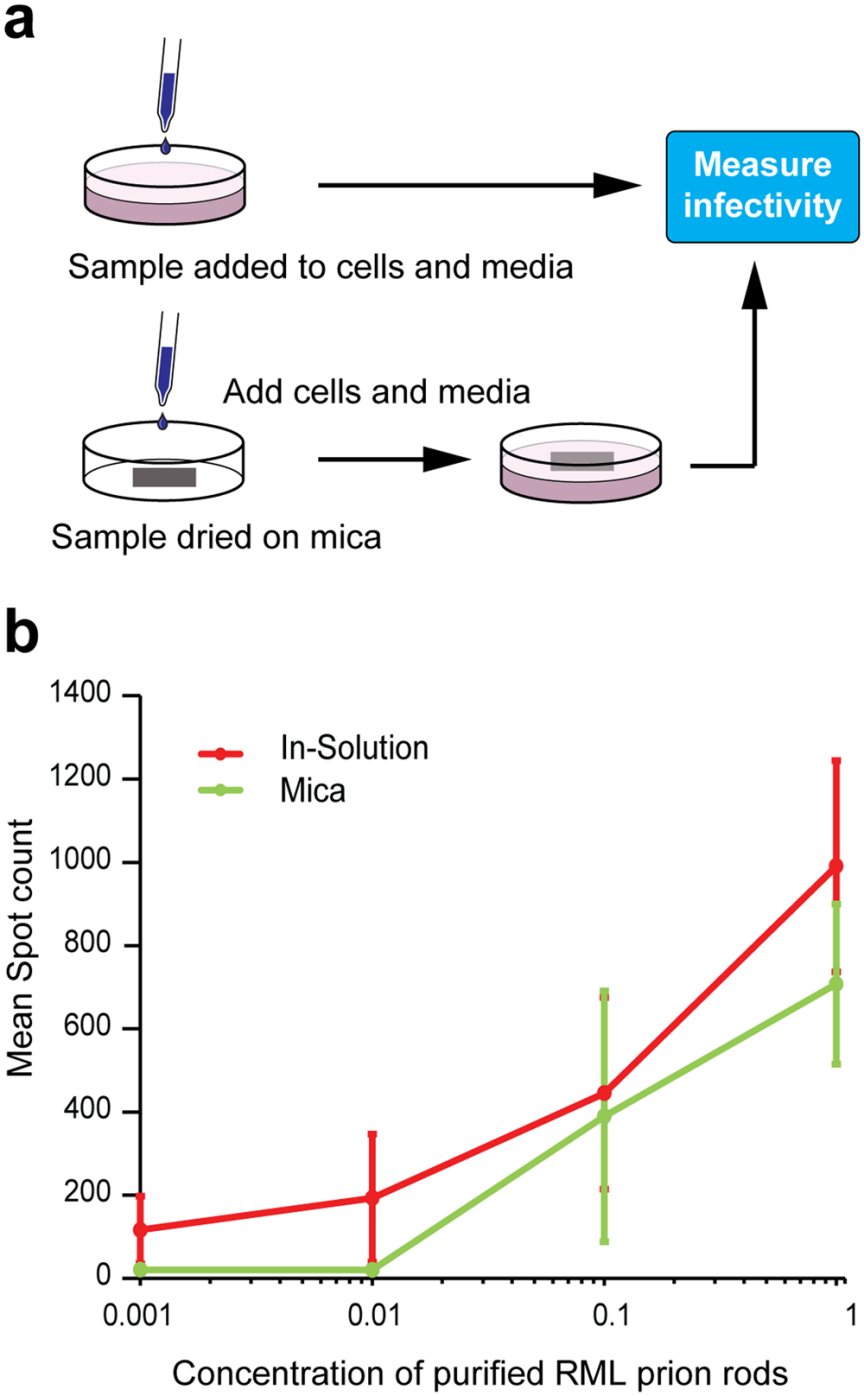
Measuring the infectivity of RML prion rods bound to mica supports for AFM. (**a**) Schematic diagram showing how prion infectivity of purified RML PrP rods was measured in cell culture. Identical aliquots of purified RML prion rods were either applied to cells as solution or dried onto sections of mica before application of cells. Infectivity was measured using methods based upon the Scrapie Cell Assay^45^. (**b**) Levels of infectivity (spot count) plotted against the concentration of purified RML prion rods (0.001–1x relative concentration range) after drying onto mica (green) or applied to cells in-solution (red). Data points show mean spot count ± SEM n = 18; 3 independent experiments using three different preparations of purified RML PrP rods. 1x relative concentration of RML prion rods in this experiment corresponds to about 2,500 intracerebral LD_50_ units in Tg20 mice. Recombinant PrP fibrils or purified P4 fractions from normal mouse brain showed no detectable prion infectivity when applied either in-solution or after drying onto mica.

In comparison to the relatively simple architecture of non-infectious recombinant PrP fibrils, infectious PrP rods isolated *ex vivo* from multiple rodent-adapted prion strains (RML and ME7 prions from mice and Sc237 prions from hamsters^8^) have a more complex structure^9^. Each PrP rod is composed of a pair of short, intertwined fibres, each with a double helical repeating substructure, separated by a distinct gap 8–10 nm in width^9^ (Figs 2b,c, 3c,d and 4a–f). This architecture is apparent when rods are imaged by negative stain EM regardless of the type of the stain used (uranyl acetate at pH 4 or NanoW at pH 6.8) (Fig. 4a,b) and is clearly observed in 3D by electron tomography^9^. We now extend our understanding of PrP rod structure by imaging unstained RML and ME7 PrP rods in their native, hydrated state by cryo-EM and also by AFM after drying onto mica. Importantly, in keeping with our finding that PrP rods when dried onto carbon-coated EM grids showed no loss of prion infectivity in cell culture^9^, PrP rods when dried onto mica substrates for AFM were also similarly infectious (Fig. 5). Findings from negative stain EM, cryo-EM and AFM therefore all report on the same authentic, biologically active, structure.

Images from both cryo-EM and AFM corresponded closely with images from the earlier negative stain EM and confirm the striking difference in the structure between the PrP rods and the single recombinant PrP fibres. In vitreous ice, RML PrP rods (Fig. 4c,d) have dimensions closely similar to those measured from negative stain EM with a width of 19.4 ± 2.3 nm (mean ± SD; n = 18) and a gap of 8–10 nm separating the paired fibres (Table 1). Repeating structural units at 6.3 ± 0.4 nm along the length of each of the paired fibres were seen by AFM (Fig. 2c) which is consistent with observations of repeating structural units in the paired fibres at intervals of 6.5 ± 0.5 nm measured from negative stain electron tomography. The overall height of the PrP rod from the mica surface (to the highest point of each paired fibre when the rod is lying flat) is 9.2 ± 0.5 nm (Fig. 2b) (Table 1) while the central gap region has a reduced height of 7.4 ± 0.8 nm where the rod appears to dip in the centre (Fig. 4e) (Table 1). Width:height ratios were consistent across all of the PrP rods examined. Importantly, imaging of RML PrP rods by AFM not only clearly defines the central 8–10 nm gap of the rod (Figs 2b,c and 4e,f) but also reveals the presence of biological material within the central gap which appears to link the paired fibres of the rod (Figs 2b,c and 4e,f). Notably this central gap material appears to have a more irregular structure along its length (Fig. 2c, dotted line) than the repeating structure seen along the length of the fibres of the rod (Fig. 2c, continuous line).

To date the adhesive properties of prion rods have not been explored. Accordingly, to examine this we used a silicon AFM tip to measure surface topography and reveal the adhesive properties of the rod (Fig. 4f). The outer surfaces of the rods appeared smooth which is consistent with our previous evidence suggesting that the PrP N-linked glycans are buried within the structure of the rod^9^. The average adhesive interaction of the rod fibres was about 70% of that of the surrounding mica surface and closely similar to that measured for recombinant PrP fibres (Table 2). In sharp contrast we found that the average adhesive force of the irregular structural material in the central gap of the rod was about 126% of that of the mica surface (Table 2). The adhesive interaction of material in the centre of the PrP rod, as compared to that of mica, is therefore nearly twice that of the rod fibres, strongly suggestive of a distinct composition.

**Figure 6:**
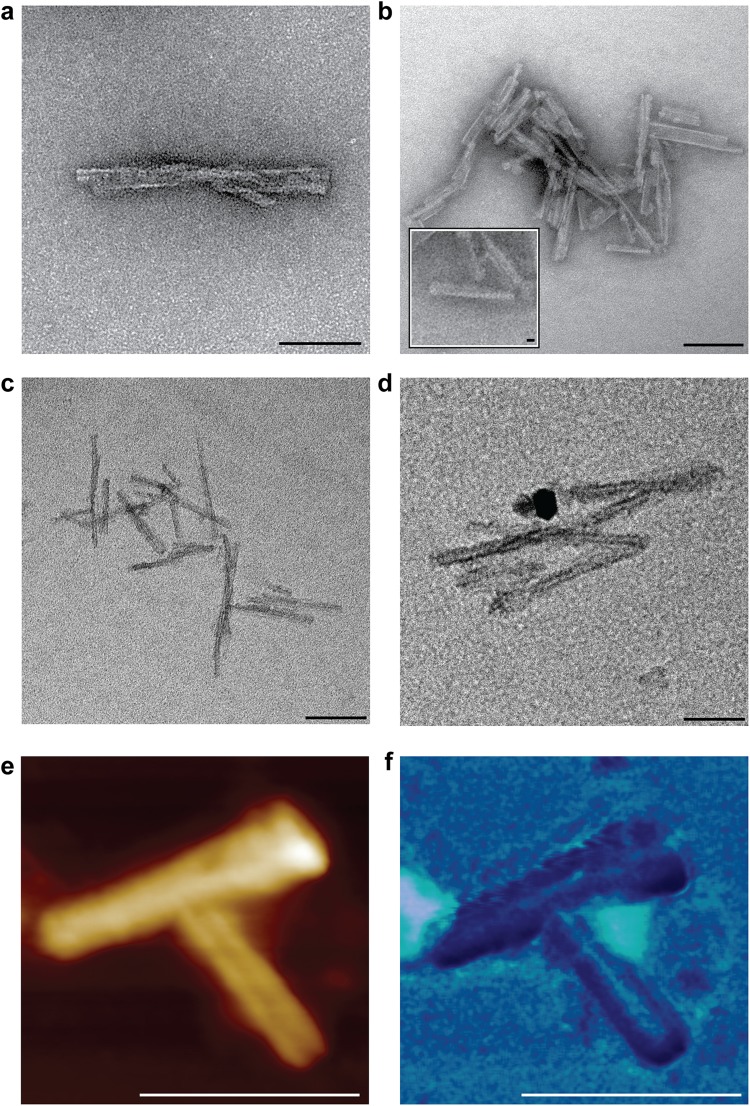
Infectious ME7 prion rods imaged by negative stain EM, cryo-EM and AFM. (**a**,**b**) Negative stain EM using (**a**) uranyl acetate at pH 4 and (**b**) NanoW at pH 6.8. (**c**,**d**) cryo-EM images in vitreous ice. (**e**,**f**) AFM scanning with high resolution tips, (**e**) height mode and (**f**) surface topography (height) adhesion force mode scanning. The adhesive force interaction of material in the central gap of the two ME7 PrP rods shown was about 1.6 times greater than that of the rod fibres. Scale bars, 100 nm panels a,b,d–f, 200 nm panel c, 10 nm inset in panel b.

In a final series of experiments we examined PrP rods from another prion strain (PrP rods purified from the brain of C57Bl/6 mice terminally infected with the ME7 prion strain^8^) by negative stain EM (Fig. 6a,b), cryo-EM (Fig. 6c,d) and AFM (Fig. 6e,f). Structural features determined by cryo-EM and AFM were entirely consistent with data from our previous analyses of ME7 prion rods by negative stain electron tomography^9^. The width of the ME7 rods measured by negative stain EM (using NanoW) was 20.5 ± 2 nm (mean ± SD; n = 18) compared to a width of 18.5 ± 2 nm (mean ± SD; n = 18) measured by cryo-EM, indicating that dehydration and staining have little effect on the dimensions of the rods. By AFM the paired fibres of the ME7 rods appeared smooth with no apparent protrusion of PrP N-linked glycans from their surface. Importantly, analogous to findings with RML prion rods, AFM revealed the presence of material in the central 8–10 nm gap of ME7 PrP rods with an adhesivity that was greater than that of the rod fibres (Fig. 6e,f). Findings for ME7 rods by all three methods therefore convincingly demonstrate that their overall architecture is closely similar to that of RML PrP rods.

## Discussion

In this study, we have examined the architecture of unstained preparations of *ex vivo* infectious PrP rods and non-infectious recombinant PrP fibrils. Our findings with cryo-EM and AFM confirm the key structural differences that we observed with negatively stained samples by electron tomography^9^ and exclude the possibility that stain distorted these structures or that staining artefacts contribute to their morphology. The basic architectures of the two assemblies are strikingly distinct. Infectious PrP rods are ~20 nm wide and are composed of two fibres (each with a double helical substructure) separated by a gap of 8–10 nm. AFM of unstained samples reveals that this central gap contains irregularly-structured material that appears to be compositionally distinct from the surface of the individual fibres. This finding is consistent with a proposed involvement of PrP N-linked glycans in linking the paired fibres of the rod^9^ as the marked protease-resistance of the rods^8,9^ is difficult to reconcile with irregularly structured material if this were comprised solely of protein. In contrast to the structural complexity of the PrP rods, non-infectious recombinant PrP fibrils are single fibres ~10 nm wide with a double helical arrangement of two protofilaments.

Based upon our previous findings^8,9^ and this study, we have defined PrP rods as the target for future high resolution studies of authentic prion structure. Recently, Wille and colleagues also sought to investigate mammalian prion structure using cryo-EM^11^, however to our surprise, the structure they described comprised a single PrP fibre ~10 nm wide composed of two protofilaments^11^ with an overall architecture closely similar to that of our non-infectious recombinant PrP fibrils. We consider that this finding is directly attributable to their use of prions isolated from the brains of transgenic mice expressing GPI-anchorless PrP (tg44 mice) infected with RML prions^11^. RML prion-infected tg44 mice replicate prions (that can be subsequently titrated in wild-type mice) but they also develop intense PrP amyloid deposits throughout their brain which are not seen in RML prion-inoculated wild-type mice^12,13^. Notably, the GPI-anchor-deficient PrP expressed in these mice is mainly non-glycosylated^12,13^, so that these mice are in effect producing a high level of what is essentially recombinant PrP along with a minor population of glycosylated PrP species. Caughey and colleagues have described two morphologically distinct PrP fibril assemblies in prion-infected tg44 mouse brain^14–16^ and inspection of their images shows clear evidence for the presence of paired fibre PrP rods (~20 nm in width) together with other single PrP fibres of ~10 nm in width. The PrP fibrils isolated from the brain of RML prion-infected tg44 mice by Wille and colleagues showed tight uniformity in width (9.55 ± 1.15 nm; n = 261)^11^ indicating that their purification protocol had resulted in preferential enrichment of these single PrP fibres. Importantly, the specific prion infectivity of their purified PrP fibre preparation (with respect to protease-resistant PrP concentration) was dramatically *reduced* compared to the RML prion-infected tg44 mouse brain homogenate used for purification. Using the original prion titration data for RML prion-infected tg44 mouse brain reported by Chesebro and colleagues^12^, the mean prion incubation periods of 153 days for RML prion-infected tg44 mouse brain homogenate and 203 days for purified PrP fibrils observed in C57Bl/6 mice by Wille and colleagues^11^ would correspond to a 10^2^–10^3^ fold reduction in infectious prion titre^12^. Collectively, based upon the published data, we conclude that RML prion-infected tg44 mouse brain propagates paired fibre PrP rods (20 nm wide) which account for the transmissible prion infectivity and structurally distinct single PrP fibres (10 nm wide) which account for the abundant PrP amyloid plaques that characterise these mice. It appears therefore that Wille and colleagues have structurally characterised the far more abundant, single fibre PrP fibrils rather than the infectious PrP rods.

Generation of GPI-anchorless PrP mice has provided significant insight into the pathogenesis of inherited forms of human prion disease in which prominent amyloid PrP plaque deposition is a major feature^12,13,17,18^. These include *PRNP* stop mutations that result in C-terminally truncated GPI-anchorless PrP isoforms and a range of *PRNP* missense point mutations some of which are associated with Gerstmann-Sträussler-Scheinker (GSS) disease phenotypes^19–22^. A common feature of some of these missense point mutations is that the expressed full-length mutant PrP forms two distinct disease-associated assemblies of misfolded PrP. One assembly forms N-terminally truncated protease-resistant fragments that correspond to those generated from classical PrP^Sc^ (PrP 27–30 see ref.^1^; analogous to that produced from *ex vivo* PrP rods^8,9^) which is enriched in brain areas showing synaptic PrP deposition, spongiform vacuolation and neurodegeneration. The other disease-associated assembly leads to smaller N- and C-terminally truncated protease-resistant fragments (typically 7–15 kDa, derived from the central region of PrP) which is associated with PrP amyloid plaques^23–31^. These distinct disease-associated PrP assembly states from GSS patients with the P102L PrP mutation transmit different phenotypes to experimental reporter mice resulting in either a lethal transmissible spongiform encephalopathy (associated with transmission of classical PrP^Sc^) or a clinically silent PrP amyloidosis (associated with the transmission of the PrP conformer generating an ~8 kDa, protease-resistant PrP fragment)^32,33^. Based upon the evidence that RML-infected tg44 mice are propagating PrP rods and structurally distinct single PrP fibres, it seems probable that both PrP rods and distinct single fibre PrP assemblies might also be propagating in some inherited prion diseases. Variation in the substructure of PrP rods or the single PrP fibrils (governed by the specific PrP missense mutation) may dictate their strain-specific transmission properties and host range via conformational selection^2,3,34,35^, while temporal and spatial differences in their accumulation (either predominantly PrP rods or single PrP fibrils within particular brain regions) might underlie the diverse clinicopathological phenotypes that are seen in family members with the same *PRNP* mutation^19,21,22,36^. Although the work of Wille and colleagues^11^ does not report the structure of infectious PrP rods, the architecture of an *ex vivo* single PrP fibril is nevertheless of major interest as the propagation of single fibrils may significantly influence clinical and pathological disease phenotypes in humans.

In summary, the structure of infectious PrP rods that we describe here readily distinguishes them from the available characterised structures of tau, amyloid-β and α-synuclein that propagate in other neurodegenerative diseases^5–7^ and from numerous other PrP aggregates proposed by others as infectious prion assembly states (for reviews see refs ^37,38^). Our finding that the characteristic 8–10 nm gap between the paired fibres of infectious PrP rods contains material with an irregular topography that is likely to be compositionally distinct to the protein surface of the outer fibres is consistent with our proposal that some of the PrP N-linked glycans are located in the gap and are contributing to the stability of the assembly, and as a consequence its infectivity^9^.

## Methods

### Research governance

Frozen brains from mice with clinical prion disease were used to generate purified prion samples. These brain samples were generated by us as part of a previous study^8^ in which work with animals was performed in accordance with licences approved and granted by the UK Home Office (Project Licences 70/6454 and 70/7274) and conformed to University College London institutional and ARRIVE guidelines. All experimental protocols were approved by the Local Research Ethics Committee of UCL Institute of Neurology/National Hospital for Neurology and Neurosurgery. Prion purification and cell based prion bioassay was conducted at UCL in microbiological containment level 3 or level 2 facilities with strict adherence to safety protocols. Work with infectious prion samples at Bristol University and Birkbeck College London was performed using dedicated sample holders and equipment with strict adherence to safety protocols. Prion samples were transported between laboratories in packaging conforming to UN 3373 Biological Substance, Category B specifications.

### Purified prions

Prions were purified from 10% (w/v) brain homogenates from terminally affected CD1 mice propagating the RML prion strain (isolate I6200) and terminally affected C57Bl/6 mice propagating the ME7 prion strain (isolate I14050) as described previously^8,9^. Brain homogenates (10% (w/v)) from uninfected CD1 mice (isolates I10340 or I14040) were used to generate control samples. Full details of the purification method have been described and we used purified P4 fractions prepared without proteinase K digestion for all experiments^8^. The method produces a recovery of ~10% of the prions present in the starting 10% (w/v) brain homogenate so that resuspension of the purified P4 pellet fraction in buffer at one tenth of the volume of the 10% (w/v) brain homogenate from which it was derived produces prion preparations whose infectivity titre matches that of the starting 10% (w/v) brain homogenate^8^. RML isolate I6200 has a prion titre of 10^7.3 ± 0.5^ (mean ± SD; n = 6) intracerebral LD_50_ units/ml when end-point titrated in Tg20 mice^8^. Concentration of purified prions or buffer exchange was achieved by centrifugation at 16,100 *g* for 30 min and resuspension of the pellet fraction into the desired volume and buffer of choice. Routine SDS-PAGE, silver staining and PrP immunoblotting to characterise purified prion preparations was performed using published procedures^8,39^.

### Recombinant PrP fibrils

Recombinant mouse PrP (*Prnp* allele a; amino acid residues 23–231) was purified from *E*. *coli* BL21(DE3) and folded in to a β-sheet rich conformation (β-PrP) in 10 mM sodium acetate/10 mM tris-acetate buffer pH 4 containing 1 mM DTT as previously described^9,10,40^. Samples were subsequently adjusted with 1 M HCl to pH 3 and a final protein concentration of 0.5 mg/ml in 10 mM sodium acetate/10 mM tris-acetate buffer, after which 100 µl aliquots were incubated for three to five months without agitation at 25 °C in sealed 1.5 ml tubes. Recombinant PrP fibrils were harvested by centrifugation at 13,000 *g* for 45 min after which pellets were resuspended in 100 µl of 10 mM sodium acetate/10 mM tris-acetate buffer pH 3.0. These stocks were stored at 25 °C.

### Negative stain electron microscopy

Full methods used have been published previously^9^. Briefly samples were loaded onto carbon-coated 300 mesh copper grids (Electron microscopy Sciences) that were glow discharged for 40 seconds using an PELCO easiGLOW™ glow discharge unit (Ted Pella Inc, USA). Samples were left to bind for 2 minutes, blotted, washed briefly in one water drop, blotted, and then stained with either NanoW stain (Nanoprobes) 2 × 1 min or 2% (w/v) uranyl acetate in water for 45 sec, then blotted and air-dried. Grids were inserted into the microscope using a dedicated sample holder for mouse prions with strict adherence to risk assessment and microbiological containment level 2 safe working practice. The sample holder was decontaminated directly after use by exposing to plasma using a Fischione 1020 plasma cleaner. Images were acquired on an FEI Tecnai T10 electron microscope (FEI, Eindhoven, NL) operating at 100 kV and recorded on a 1 k × 1 k charged couple device (CCD) camera (Gatan) at a nominal magnification of 44,000 with a pixel size of 3.96 Å. Fibril dimensions were measured in ImageJ^41^ and IMOD^42^. Because of their helical twist, prion rods and recombinant PrP fibrils viewed on a surface alternate between wider, face-on and narrower, edge-on views of the structure. The dimensions reported here were measured on the widest parts of the fibrils.

### Cryo-electron microscopy

We examined purified prion rods (typically resuspended in 1/100 of the volume of 10% (w/v) brain homogenate from which they were derived) and recombinant PrP fibrils (stock preparations diluted 5-fold in 10 mM sodium acetate/10 mM tris-acetate buffer pH 3.0). Protein samples were sonicated for 20 sec in a Sonicator 3000 (Misonix) at 40 W at 4 °C and then mixed with Protein A-10 nm gold solution (Electron Microscopy Sciences) (pre-sonicated for 5 sec as above) at a ratio of 1:25 (v/v). 3 µl of sample was loaded onto carbon-coated 300 mesh copper or gold grids (Electron Microscopy Sciences) that had been glow discharged for 1 min using a PELCO easiGLOW™ glow discharge unit (Ted Pella Inc, USA) and allowed to bind for 1.5 min. Grids were then blotted for 4–7 sec prior to freezing in liquid ethane using a manual plunging device after which the grids were stored in liquid nitrogen. Samples were imaged using a dedicated Gatan cryo-transfer holder (model 626) on an FEI Tecnai T12 electron microscope (FEI, Eindhoven, NL) operating at 120 kV and recorded using a US4000 CCD camera (Gatan) typically using 1 to 3 µm defocus and a nominal magnification of 42,000 with a pixel size of 4.1 Å. After use, the holder was decontaminated using a Fischione 1020 plasma cleaner. Fibril dimensions were measured in ImageJ^41^ and IMOD^42^. Fibril and rod dimensions were measured on the widest regions of the fibrils, as for the negative stain EM.

### Atomic force microscopy

Prior to loading onto mica, purified prions (typically resuspended in 1/25 of the volume of 10% (w/v) brain homogenate from which they were derived) and recombinant PrP fibril samples (0.5 mg ml^−1^ protein) were washed once with water to reduce the concentration of contaminating salts and detergents. This was done by resuspending 40 µl of each sample in 500 µl of ultrapure water and centrifugation at 16,100 *g* for 30 min after which the supernatant was discarded and the bottom of the tube containing the sample resuspended in 40 µl ultrapure water. Samples were then sonicated briefly for 10 sec using a Sonicator 3000 (Misonix) at 40 W at 4 °C after which 1 µl of sample was dispensed onto a freshly cleaved mica surface and air-dried. Samples were investigated at room temperature using a Multi-mode VIII microscope with Nanoscope V controller and utilising a non-resonant, PeakForce feedback mechanism. In addition, a fast scanning head unit was employed with SCANASYST-AIR-HR cantilevers (Bruker) with nominal spring constant, k of 0.4 N/m and nominal tip radius 2 nm for imaging large areas (up to 50 µm × 50 µm) in order to identify candidates for high resolution investigation. SCANASYST-FLUID+ cantilevers (Bruker) with a nominal spring constant, k of 0.7 N/m and nominal tip radius <2 nm were used for high resolution imaging of individual PrP rods or recombinant PrP fibrils and also for mapping of adhesion interactions across the surface of each. Adhesion interaction forces were extracted in real-time from the force curves collected as part of the PeakForce control mechanism. Whilst the cantilever type was kept constant between samples, fresh cantilevers were used for each to ensure the highest possible topographic resolution was obtained. When comparing adhesion interaction forces for different samples it was therefore necessary to negate possible influences of small differences in tip radius through normalising to that of the mica background consistent across all samples^43,44^. Data was analysed using Nanoscope Analysis software (Bruker). Dimensions reported by AFM in Table 1 were determined from 8 RML prion rods or 6 recombinant PrP fibrils. Both assemblies vary in height and width according to their helical twist, and the dimensions reported in Table 1 were determined from the widest regions, as for the EM. Adhesion force interaction measurements were similarly determined from 8 RML prion rods or 6 recombinant PrP fibrils taking multiple measurements along their length as reported in Table 2. Statistical analyses of mean adhesion force interaction values were performed using Graphpad Instat software with p values calculated using an unpaired t test with Welch correction.

### Mica-bound prion infectivity assay

To measure RML prion infectivity bound to mica, samples of mica (Agar Scientific) were cut into 0.5 cm^2^ pieces and their surface freshly cleaved using Scotch Magic Tape. 1 µl aliquots of sample, either recombinant PrP fibrils at protein concentrations of 0.5, 0.05, 0.005 and 0.0005 mg ml^−1^, or purified RML prions (initially resuspended in a volume equivalent to that of the starting 10% (w/v) brain homogenate from which they were derived and 10-fold serial dilutions thereof), were applied to the mica surface and air-dried. Mica sections were then placed in the bottom of the wells of tissue culture plates (Nunc 12-well × 2 ml polystyrene multidish, Capitol Scientific) followed by the addition of 2.5 ml of PK1/11 cell suspension (80 cells µl^−1^) in OFCS (OptiMEM, 10% FCS, penicillin and streptomycin) to give a total of 200,000 cells per well. In parallel, replicate tissue culture plates were prepared in which 1 µl of each purified prion or recombinant PrP sample was applied as solution to wells containing PK1/11 cells (as above). For both conditions cells were then incubated for 3 days. Cells growing on mica were harvested by transferring each mica section to a well of a new tissue culture plate followed by washing with 2 × 500 µl OFCS medium to remove cells from the mica surface, after which the wash fractions were pooled. Cells from the replica plates in which samples had been applied as solution were thoroughly resuspended in 1 ml OFCS medium. All recovered cell suspensions were then adjusted to a density of 16 cells µl^−1^ with OFCS media and 250 µl aliquots (4000 cells) transferred to 6 wells of a new 96 well tissue culture plate (96 Well Clear Flat Bottom Polystyrene TC-Treated Microplate, Corning) and grown to confluence for 7 days. Subsequently cells were split at a 1:8 dilution into fresh OFCS media and grown to confluence for three days and then passaged in a standard Scrapie Cell Assay format (three successive cycles of 1:8 splits with three days incubation between splits) before transferring aliquots of the cells to ELISPOT plates for analysis of the number of cells containing proteinase K-resistant PrP “spot count”^8,45,46^. As a negative control, purified P4 samples from non-infected CD1 mouse brain were analysed in an identical fashion. We analysed three different purified P4 preparations from RML-infected CD1 mouse brain using these methods and in each case the prion titre of the P4 samples (as determined using the standard Scrapie Cell Assay^8,46^) was entirely consistent with our published findings^8^. Preliminary experiments in which freshly cleaved blank mica sections were incubated with PK1/11 cells showed no deleterious effects on cell health or viability.

## Data Availability

The datasets generated and analysed during the current study are available from the corresponding author on reasonable request.
